# The six-minute walk test in patients with pulmonary sarcoidosis

**DOI:** 10.4103/1817-1737.49414

**Published:** 2009

**Authors:** Esam H. Alhamad

**Affiliations:** *Department of Medicine, Division of Pulmonary Medicine, King Khalid University Hospital, King Saud University, Riyadh, Saudi Arabia*

**Keywords:** Functional status, pulmonary sarcoidosis, 6-min walk test

## Abstract

**BACKGROUND:**

The 6-min walk test (6MWT) is a useful tool to assess prognosis and functional impairment in various pulmonary diseases.

**AIMS:**

To evaluate functional capacity during various stages of pulmonary sarcoidosis and develop a scoring system clinical radiological physiological score (CRP) that can potentially be used to assess the functional status among patients with sarcoidosis.

**MATERIALS AND METHODS:**

We performed a retrospective study on 26 patients diagnosed with pulmonary sarcoidosis from 2001 to 2007. All patients completed the 6MWT. The parameters assessed during the test included spirometry, arterial blood gas, 6-min walk distance (6MWD), Borg dyspnea score, and initial and end oxygen saturation.

**RESULTS:**

Females covered a significantly shorter distance than males (343 m (223–389) vs. 416.5 m (352–500); *P* < 0.0001). In addition, females had a significantly lower SpO_2_ at the end of the 6MWT than males (90.5 (61–99) vs. 96 (75–98); *P* < 0.03). The 6MWD was inversely correlated with the final Borg score (ρ = −0.603, *P* = 0.004) and the CRP score (ρ = −0.364, *P* = 0.047) and positively correlated with forced expiratory volume in 1 s (FEV_1_) % (ρ = 0.524, *P* = 0.006) and forced vital capacity (FVC) % (ρ = 0.407, *P* = 0.039).

**CONCLUSIONS:**

Female gender, FEV_1_%, final Borg score, FVC%, CRP score, and SpO_2_ at the end of the 6MWT are associated with reduced 6MWD. It appears that Saudi patients diagnosed with sarcoidosis have a markedly reduced walking distance compared with other races. The effect of race and ethnicity and the utility of the CRP score as a potential marker to assess functional status require further exploration.

Sarcoidosis is a systemic granulomatous disease of unknown etiology that is highly variable and characterized by diverse organ system manifestations. The lungs are affected in the majority of the patients. Dry cough, dyspnea on exertion, and vague retrosternal chest pain are common pulmonary manifestations. Constitutional symptoms, such as fever, fatigue, general weakness, and weight loss, occur in about one-third of the patients with sarcoidosis. While symptomatic muscle involvement is rare, subclinical skeletal muscle involvement is observed in up to 80% of the sarcoidosis patients[1] These nonspecific symptoms are disabling for patients, causing impairment in functional capacity and quality of life. The 6-min walk test (6MWT) is widely used to assess the functional status of patients with various pulmonary diseases, including idiopathic pulmonary fibrosis, chronic obstructive lung disease (COPD), and pulmonary hypertension, and nonpulmonary causes, such as chronic liver disease, multiple sclerosis, and chronic heart failure.[2–7] Because the test is simple, inexpensive, reproducible, and well received by patients, it has replaced standard cardiopulmonary exercises for the evaluation of lung disease.[3,8] Kabitz *et al.*[9] showed a significant reduction in the 6-min walk distance (6MWD) achieved by 24 male patients diagnosed with sarcoidosis, compared with the control group. The investigators additionally identified twitch mouth pressure and Borg dyspnea scale as the strongest predictors of 6MWD performance. Another study attempted to identify prognostic factors for advanced sarcoidosis patients listed for lung transplantation.[10] The walking distance among patients who eventually died was generally reduced, although the results did not reach statistical significance. Baughman *et al.*[11] observed a decrease in the 6MWD in the majority of the sarcoidosis patients. Factors that were significantly correlated with reduced 6MWD included forced vital capacity (FVC), oxygen saturation with exercise, and self-reported respiratory health.

This retrospective study was conducted to explore the hypothesis that Saudi patients diagnosed with sarcoidosis might have other variables that affect the 6MWD compared with other races. The aims of our study were to (1) assess functional capacity during various stages of pulmonary sarcoidosis, (2) identify variables that correlate with a reduction in walking distance, and (3) develop a scoring system (the clinical radiological physiological score (CRP) that can potentially be used to assess the functional status among patients with sarcoidosis.

To our knowledge, the 6MWT has not been applied to evaluate sarcoidosis patients in Saudi Arabia.

## Materials and Methods

### Study population

The study involved 26 patients diagnosed with pulmonary sarcoidosis from January 2001 to January 2007 attending the outpatient pulmonary clinics at the King Khalid University Hospital (Riyadh, Saudi Arabia). The investigation was approved by the Ethics Committee of the hospital. Sarcoidosis was diagnosed based on the latest ATS/ERS/WASOG statements.[12] Patients displaying evidence of mycobacterial or fungal infections and those with a history of ingestion of drugs or agents causing granulomatous lung disease were excluded from the study. Relevant clinical data, age, and gender were recorded.

Chest radiography (CXR) was staged according to Scadding: Stage 0 (no radiographic abnormalities), stage 1 (bilateral hilar adenopathy without parenchymal abnormalities), stage 2 (bilateral hilar adenopathy with interstitial parenchymal infiltrates), stage 3 (interstitial parenchymal infiltrates without hilar adenopathy), and stage 4 (pulmonary fibrosis).[12] The treating pulmonary physician determined the radiographic stage in all patients.

The following procedures were performed to obtain diagnostic histological specimens: Bronchoscopy (transbronchial or bronchial biopsy), mediastinoscopy, video-assisted thoracoscopic surgery, open lung biopsy, and peripheral lymph node biopsy.

The treatment regimens for sarcoidosis were grouped as follows: None (no treatment), prednisone, prednisone and azathioprine, and prednisone and methotrexate. Treatment decisions were based on the opinion of the treating physician.

### Physiologic testing and the 6MWT

The pulmonary functions of all patients were evaluated on the day of the 6MWT. The time interval between diagnosis and the 6MWT did not exceed 4 weeks. Using the American Thoracic Society recommendations,[13,14] spirometry, plethysmographic lung volumes, and single-breath diffusion capacity for carbon monoxide (DLco) (671178; Erich Jaeger Master Screen PFT, GmbH D-9204 Hoechben, Germany) were measured. FVC, forced expiratory volume in 1 s (FEV_1_), and total lung capacity were determined and values were expressed as a percentage of the predicted values, calculated according to gender, weight, and age. DLco measurements were performed using the single- breath technique and results were corrected for the patient's level of hemoglobin. Arterial blood gas sampling (Rapid lab 865; Bayer, Plymouth, UK) included arterial potential of hydrogen (pH), partial pressure of oxygen (PaO_2_), partial pressure of carbon dioxide (PaCO_2_), and oxygen saturation measurements.

Patients were instructed to walk in a hallway for 6 min according to the American Thoracic Society guidelines.[15] All patients had a normal resting oxygen saturation (SpO_2_) at the beginning of the walk test. Heart rate, blood pressure, oxygen saturation, and Borg dyspnea index[16] were recorded at the beginning and at the end of the 6-min walk. The total distance walked in meters was documented at the end of the test. Patients using oxygen therapy were excluded from the study. This served to standardize the data and eliminate supplemental oxygen as a confounding factor in the determination of variables that correlate with a reduction in walking distance.

### CRP score

CRP was calculated as the sum of all the scores. Individual symptoms, such as cough, dyspnea, and weight loss, if present, were assigned a score of 1. Radiological staging was scored as follows: Stage 0=0, stage 1=1, stage 2=2, stage 3=3, and stage 4=4, physiological study scored as the presence of obstructive, restrictive, or mixed defects (=1), reduced DLco =1, and partial pressure of oxygen (PaO_2_) <70 mmHg =1). For example, a patient with symptoms of cough and dyspnea, radiographic stage 4, mixed obstructive and restrictive defects, low DLco, and PaO_2_ = 68 mmHg was scored as 9 (1 + 1 + 4 + 1 + 1 + 1 = 9).

### Statistical analysis

Data were collected with Microsoft Excel and analyzed using the Statistical software package for Social Sciences (SPSS version 11.5; SPSS Inc., Chicago, IL, USA). Descriptive statistics (mean, standard deviation, median (interquartile range), and proportion) were applied to summarize the data. The 6MWD was compared with various parameters using nonparametric testing, including Spearman rank correlation (ρ) and Mann– Whitney *U*-test; *P* < 0.05 was considered significant. Sensitivity and specificity were used to evaluate test criteria “CRP.”

## Results

### Patient characteristics

Demographic and clinical data are summarized in Table 1. The results of pulmonary function studies and characteristics of patients, along with the 6MWDs of different patient groups, are listed in Table 2. The mean age at presentation was 43.5 (range 24–84) and the male-to-female ratio was approximately 1:1. The mean body mass index was higher for females than males (32.6 + 7.6 and 28.8 + 2.7 kg/m^2^, respectively), but this difference was not statistically significant. Pulmonary function analyses disclosed that 23% of the patients were normal, 23% had an obstructive ventilatory defect, 34.6% were restrictive, 3.8% had mixed defects, and 15.3% displayed reduced DLco alone.

**Table 1:** Demographic and clinical characteristics of sarcoidosis patients

Characteristics	Values
Age (years)	43.5 + 11.8
Sex: Male/Female	12/14
Presenting symptoms	
Dyspnea	23 (88.5)
Cough	22 (84.6)
Weight loss	8 (30.8)
Comorbid illness	
Diabetes mellitus	5 (19.2)
Hypertension	3 (11.5)
Diagnostic procedure	
TBB	14 (53.8)
Mediastinoscopy	3 (11.5)
Open lung biopsy	3 (11.5)
Other	6 (23)
Treatment	
None	6 (23)
Prednisone	14 (53.8)
Prednisone + azathioprine	3 (11.5)
Prednisone + methotrexate	3 (11.5)

Values are presented as means + SD, or no. (%); TBB - transbronchial biopsy

**Table 2:** Data from pulmonary function studies and patient characteristics, as well as 6MWD for different patient groups

Characteristics	Values	6MWD (m)
Total patients, no.	26	364 (223–500)
Gender		
Men	12 (46.2)	416.5 (352–500)
Women	14 (53.8)	343 (223–389)
Receiving prednisone therapy		
Yes	20 (76.9)	356 (223–463)
No	6 (23.1)	389 (332–500)
FVC, % predicted	80.4 (17.7–102)	
FEV_1_, % predicted	83.4 (21.8–110)	
TLC, % predicted	80.5 (28.7–98.3)	
DLco, % predicted	45.9 (19.8–84.7)	
PaO_2_ (mmHg)	71 (26.6–94)
Radiographic stage		
0	2 (7.7)	411.5 (360–463)
1	9 (34.6)	389 (275–500)
2	11 (42.3)	389 (270–436)
4	4 (15.4)	356 (223–364)

Values are expressed as median (interquartile range) or no. (%); 6MWD, 6-min walk distance in meters; FVC, forced vital capacity; FEV_1_, forced expiratory volume in 1 s; TLC, total lung capacity; DLCO, diffusion capacity for carbon monoxide; PaO_2_, partial pressure of oxygen

### 6MWT

The median 6MWD for the entire cohort was 364 m. Seven patients (26.9%) accomplished 6MWD >400 m, 16 (61.5%) covered 300–400 m, and three (11.5%) walked < 300 m. Females covered a significantly shorter distance than males (343 m (223–389) vs. 416.5 m (352–500); *P* < 0.0001). While patients diagnosed with higher chest radiographic stage generally displayed lower 6MWD, the correlation between radiographic stage and 6MWD was not statistically significant (*P* > 0.05). Patients receiving prednisone therapy tended to achieve shorter 6MWDs than those in the other treatment groups (361.4 m vs. 400.2 m), but not to a level of statistical significance (*P* > 0.05).

**Figure 1:**
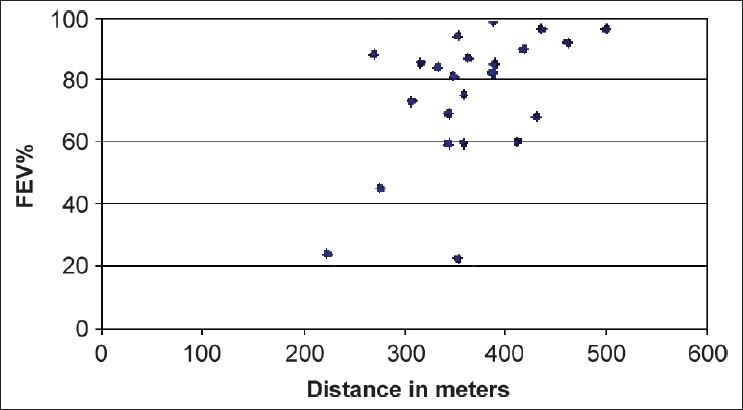
Relationship between the 6-min walk distance and forced expiratory volume in 1 s percent. A significant positive correlation was observed (ρ = 0.524, *P* = 0.006)

**Figure 2:**
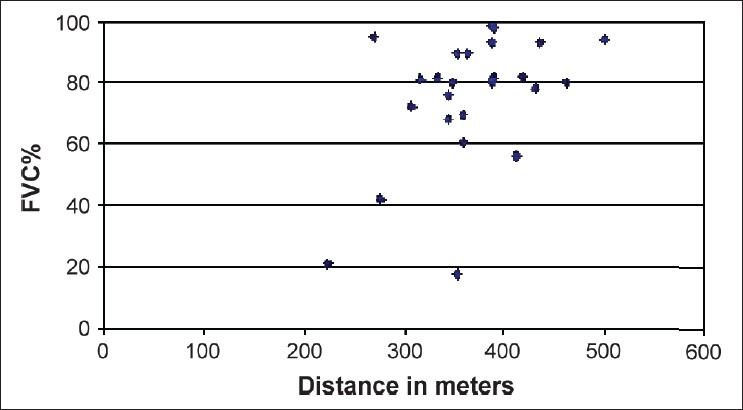
Relationship between the 6-min walk distance and forced vital capacity %. A significant positive correlation was observed (ρ = 0.407, *P* = 0.039)

**Figure 3:**
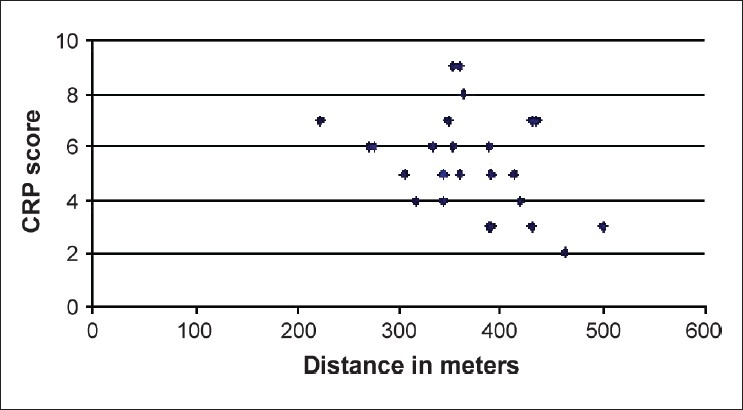
Relationship between the 6-min walk distance and the clinical radiological physiological score. The two variables were significantly correlated (ρ = −0.364, *P* = 0.047)

The 6MWD data were compared with pulmonary function analyses, arterial blood gases, Borg score, and oxygen saturation at the beginning and at the end of the 6MWT. Females had a significantly lower SpO_2_ at the end of the 6MWT than males (90.5 (61–99) vs. 96 (75–98); *P* < 0.03). The 6MWD was positively correlated with FEV_1_ % [Figure 1; ρ = 0.524, *P* = 0.006] and FVC% [Figure 2; ρ = 0.407, *P* = 0.039] and inversely correlated with the final Borg score (ρ = -0.603, *P* = 0.004) and CRP score [Figure 3; ρ = −0.364, *P* = 0.047].

A CRP score >3 yielded a sensitivity and specificity of 100% and 35%, respectively.

## Discussion

There is no single specific presentation for sarcoidosis. However, more than 90% of the patients with sarcoidosis have lung involvement,[12] commonly presenting respiratory symptoms such as cough and dyspnea. The rate of cough at the time of diagnosis varies between regions, from 3% of the patients in Japan[17] to 100% in another series.[18] Moreover, the mode of presentation differs between countries. For instance, Japanese patients are more likely to present with ocular symptoms whereas respiratory symptoms and erythema nodosum are common clinical presentations in Finland.[17] In the current cohort, dyspnea and cough were the most common complaints, consistent with previous reports on Arab patients with sarcoidosis.[19–21] Constitutional symptoms, such as weight loss, were observed in 30.8% of the patients. Earlier studies found that 26.8–60% of the sarcoidosis patients experienced weight loss.[19,22] This wide range is possibly influenced by differences in reporting and methodology among the studies. Chest radiograph findings play a crucial diagnostic role and aid in defining prognosis. Abnormalities are identified in up to 95% of the patients with sarcoidosis.[23] Radiographic stage 2 was the predominant finding (42.3%) in the present study, in contrast to previous reports in other regions where stage 1 was the most common radiographic abnormality.[17,24] Stage 2 appears more common among Middle Eastern patients with sarcoidosis than in other parts of the world.[19–21] One potential explanation for this finding is that because tuberculosis is endemic in the Middle East, many sarcoidosis patients are presumptively treated with antituberculous drugs and only referred if they do not respond to treatment. Pulmonary function analysis in the present study revealed normal, obstructive, restrictive, mixed defects, or reduced DLco alone. Restrictive abnormalities were the most frequent in our cohort of patients. Abnormalities in pulmonary function tests (PFTs) were detected in 20% of the patients with stage 1 disease, but in 40–> 70% of the patients with stage 2, 3, or 4 disease.[12] Interestingly, two patients in the present study with stage 0 disease exhibited spirometry abnormalities as well as decreased DLco, consistent with previous reports.[25,26] Exercise intolerance is commonly encountered in sarcoidosis patients.[27–30] Such limitations are due to several factors, including dyspnea, insufficient heart rate response, decreased arterial oxygen tension during exercise, as well as respiratory and skeletal muscle weakness.[9,27–31] Patients with sarcoidosis may have exercise limitations despite normal PFTs.[28,29] Miller *et al.* showed that 50% of the sarcoidosis patients with normal PFT and normal DLco exhibit abnormalities in ventilatory exercise response, suggesting an occult state of lung impairment that may contribute to exercise limitations.[29] This finding was further confirmed by another study.[28]

Few studies have examined the 6MWD in patients with sarcoidosis.[9–11,31,32] To our knowledge, only one report has assessed the correlations between 6MWD, pulmonary function, and CXR.[11] Several factors, including skeletal muscle weakness, oral corticosteroid treatment, myositis, pulmonary hypertension, respiratory status, depression, fatigue, and high circulating levels of tumor necrosis factor-α (TNF-α) significantly reduce the 6MWD in sarcoidosis.[1,11,31–37] In the current investigation, 6MWD was significantly affected by gender, pulmonary function, dyspnea, CRP score, and SpO_2_ at the completion of the 6MWT. The total distance achieved by the entire cohort was markedly reduced. In particular, women presented significantly shorter 6MWD compared with men. Kadikar *et al.* performed a retrospective study to determine the optimal timing for lung transplantation for various lung diseases but did not include patients with sarcoidosis. They showed that a 6MWD of <400 m represents a marker for transplant evaluation.[38] However, one would need to consider the effects of race and ethnicity on the normal predicted distance. Poh *et al.* showed that the 6MWD is shorter for Singaporean Chinese individuals than those of Caucasian descent.[39] A sedentary lifestyle and inactivity leading to physical deconditioning play a major role in lower 6MWDs among Saudi subjects. Al-Nozha *et al.* conducted a large national epidemiological health survey in 17,395 Saudi males and females aged 30–70 years. The prevalence of inactivity was 98.1% in women and 93.9% in men.[40] Thus, encouraging patients to exercise and perhaps enrolling into pulmonary rehabilitation programs should improve health status and skeletal muscle function, reduce the symptoms of fatigue, and lower the circulating levels of the pro-inflammatory cytokine, TNF-α.[41]

CRP is a scoring system that fits a number of variables and attempts to correlate with 6MWD. The advantage of this scoring method is that it incorporates clinical symptoms as well as radiographic staging, along with physiological testing, and thus perhaps reflects the disease more accurately than individual lung function indices. To our knowledge, such a scoring system has not been tested in sarcoidosis patients until now. More complex scoring methods have been utilized for patients with idiopathic pulmonary fibrosis and present useful tools in assessing disease severity and predicting survival.[42–44] CRP significantly correlated with walking distance and potentially appears as an effective prognostic tool in patients diagnosed clinically with sarcoidosis. Further studies on a large scale are warranted to explore these effects.

The present study has several limitations. Firstly, this investigation involved a retrospective review of patients. There is, by definition, a selection bias present, because the study was performed at a tertiary hospital to which patients with severe complaints and advanced stages of sarcoidosis are more likely to be referred. Moreover, the study cohort included patients who attended the pulmonary clinic, where extrapulmonary cases are often missed, although we did not aim to examine the prevalence of pulmonary sarcoidosis but correlate 6MWD with various stages of the disease.

In conclusion, 6MWD was abnormal in the majority of the sarcoidosis patients. Several factors contributed to reduced 6MWD, including gender, FEV_1_%, final Borg score, FVC%, CRP score, and SpO_2_ at the end of the 6MWT. Use of 6MWD should thus allow better assessment of the health status and follow-up response to treatment. It appears that Saudi patients diagnosed with sarcoidosis have markedly reduced walking distance compared with other races. A larger sample size would be required to explore the effect of race and ethnicity on the 6MWD among patients with sarcoidosis. The utility of the CRP score as a potential useful marker to assess functional status requires further exploration.
